# Dietary patterns and type 2 diabetes among Ghanaian migrants in Europe and their compatriots in Ghana: the RODAM study

**DOI:** 10.1038/s41387-018-0029-x

**Published:** 2018-04-25

**Authors:** Cecilia Galbete, Mary Nicolaou, Karlijn Meeks, Kerstin Klipstein-Grobusch, Ama de-Graft Aikins, Juliet Addo, Stephen K. Amoah, Liam Smeeth, Ellis Owusu-Dabo, Joachim Spranger, Charles Agyemang, Frank P. Mockenhaupt, Erik Beune, Karien Stronks, Matthias B. Schulze, Ina Danquah

**Affiliations:** 1 Department of Molecular Epidemiology, German Institute of Human Nutrition Potsdam-Rehbruecke (DIfE), Nuthetal, Germany; 2 Department of Public Health, Academic Medical Center, University of Amsterdam, Amsterdam Public Health Research Institute, Amsterdam, The Netherlands; 3 Julius Global Health, Julius Center for Health Sciences and Primary Care, University Medical Center Utrecht, Utrecht University, Utrecht, The Netherlands; 4 Division of Epidemiology & Biostatistics, School of Public Health, Faculty of Health Sciences, University of the Witwatersrand, Johannesburg, South Africa; 5 Regional Institute for Population Studies, University of Ghana, Legon-Accra, Ghana; 6 Department of Non-communicable Disease Epidemiology, Faculty of Epidemiology and Population Health, London School of Hygiene and Tropical Medicine, London, UK; 7 Institute of Tropical Medicine and International Health, Charité - Universitaetsmedizin Berlin, Corporate Member of Freie Universität Berlin, Humboldt-Universität zu Berlin, and Berlin Institute of Health, Berlin, Germany; 8 Department of Global and International Health, School of Public Health, Kwame Nkrumah University of Science and Technology, Kumasi, Ghana; 9 Department of Endocrinology and Metabolic Diseases, Charité - Universitaetsmedizin Berlin, Corporate Member of Freie Universität Berlin, Humboldt-Universität zu Berlin, and Berlin Institute of Health, Berlin, Germany; 10 Institute for Social Medicine, Epidemiology and Health Economics, Charité - Universitaetsmedizin Berlin, Corporate Member of Freie Universität Berlin, Humboldt-Universität zu Berlin, and Berlin Institute of Health, Berlin, Germany

## Abstract

**Background/objectives:**

We aimed to study the associations of dietary patterns (DPs) with type 2 diabetes (T2D) among Ghanaian adults.

**Subjects/methods:**

In the multi-centre, cross-sectional RODAM (Research on Obesity and Diabetes among African Migrants) study (*n* = 4543), three overall DPs (“mixed”, “rice, pasta, meat and fish,” and “roots, tubers and plantain”) and two site-specific DPs per study site (rural Ghana, urban Ghana and Europe) were identified by principal component analysis. The DPs–T2D associations were calculated by logistic regression models.

**Results:**

Higher adherence to the “rice, pasta, meat and fish” DP (characterized by legumes, rice/pasta, meat, fish, cakes/sweets, condiments) was associated with decreased odds of T2D, adjusted for socio-demographic factors, total energy intake and adiposity measures (odds ratio (OR)_per 1 SD_ = 0.80; 95% confidence interval (CI) = 0.70–0.92). Similar DPs and T2D associations were discernible in urban Ghana and Europe. In the total study population, neither the “mixed” DP (whole grain cereals, sweet spreads, dairy products, potatoes, vegetables, poultry, coffee/tea, sodas/juices, olive oil) nor the “roots, tubers and plantain” DP (refined cereals, fruits, nuts/seeds, roots/tubers/plantain, fermented maize products, legumes, palm oil, condiments) was associated with T2D. Yet, after the exclusion of individuals with self-reported T2D, the “roots, tubers and plantain” DP was inversely associated with T2D (OR_per 1 SD_ = 0.88; 95% CI = 0.69–1.12).

**Conclusion:**

In this Ghanaian population, DPs characterized by the intake of legumes, fish, meat and confectionery were inversely associated with T2D. The effect of a traditional-oriented diet (typical staples, vegetables and legumes) remains unclear.

## Introduction

The International Diabetes Federation (IDF) estimated in the 7th Atlas edition (2015) that about 75% of the adults with diabetes mellitus are living in low-income and middle-income countries^1^. Regarding the situation in the IDF-African region, there were more than 14 million people with diabetes mellitus in 2015, and this number will be more than doubled by 2040. This burden extends beyond Africa: ethnic minorities and migrant populations in Europe have shown to be disproportionately affected by type 2 diabetes mellitus (T2D)^2–5^.

Diet constitutes one of the major modifiable factors of T2D^6, 7^. In this regard, both, urbanization in sub-Saharan Africa and international migration, can lead to changes in the dietary habits, from a more traditional diet to a more westernized one. In the case of urbanization, this is due to the introduction of new food-related markets^8^. Upon migration from Africa to Europe, individuals relocate to a completely new environment, thus facing a process comparable to a sudden urbanization^9^. The study of diet is complex since an individual’s dietary intake is not composed of single foods or nutrients but consists of a combination of many nutrients and foods that may act synergistically. Thus, in the past years, nutritional epidemiology has paid attention to the study of dietary patterns (DPs)^10, 11^. In a previous work, we deeply described food consumption and explored DPs in Ghanaian migrants in Europe and their compatriots in Ghana within the context of the large RODAM (Research on Obesity and Diabetes among African Migrants) study^12^. We identified three overall DPs; DP adherence was mainly driven by study site. In Europe, the DP named “mixed” (whole grain cereals, sweet spreads, dairy products, potatoes, vegetables, poultry, coffee and tea, sodas and juices, olive oil, margarine, condiments) was the most prominent. In urban Ghana, the DP called “rice, pasta, meat and fish” (dairy products, red meat, processed meat, eggs, legumes, rice and pasta, fish, meaty mixed dishes, cakes and sweets and condiments) prevailed. And in rural Ghana, the “roots, tubers and plantain” DP (refined cereals, fruits, nuts and seeds, roots, tubers and plantain, fermented maize products, legumes and palm oil) predominated. On the background of previously identified DPs, the present work aimed at investigating the associations of these DPs with T2D in the total RODAM study population. Moreover, due to the high site-specificity of the previously described DPs as well as the observed differences in T2D prevalence across rural Ghana, urban Ghana and Europe^2^, we also aimed at constructing DPs in the three different locations and at examining their relationships with T2D.

## SUBJECTS AND METHODS

### Study design and population

**Fig. 1:**
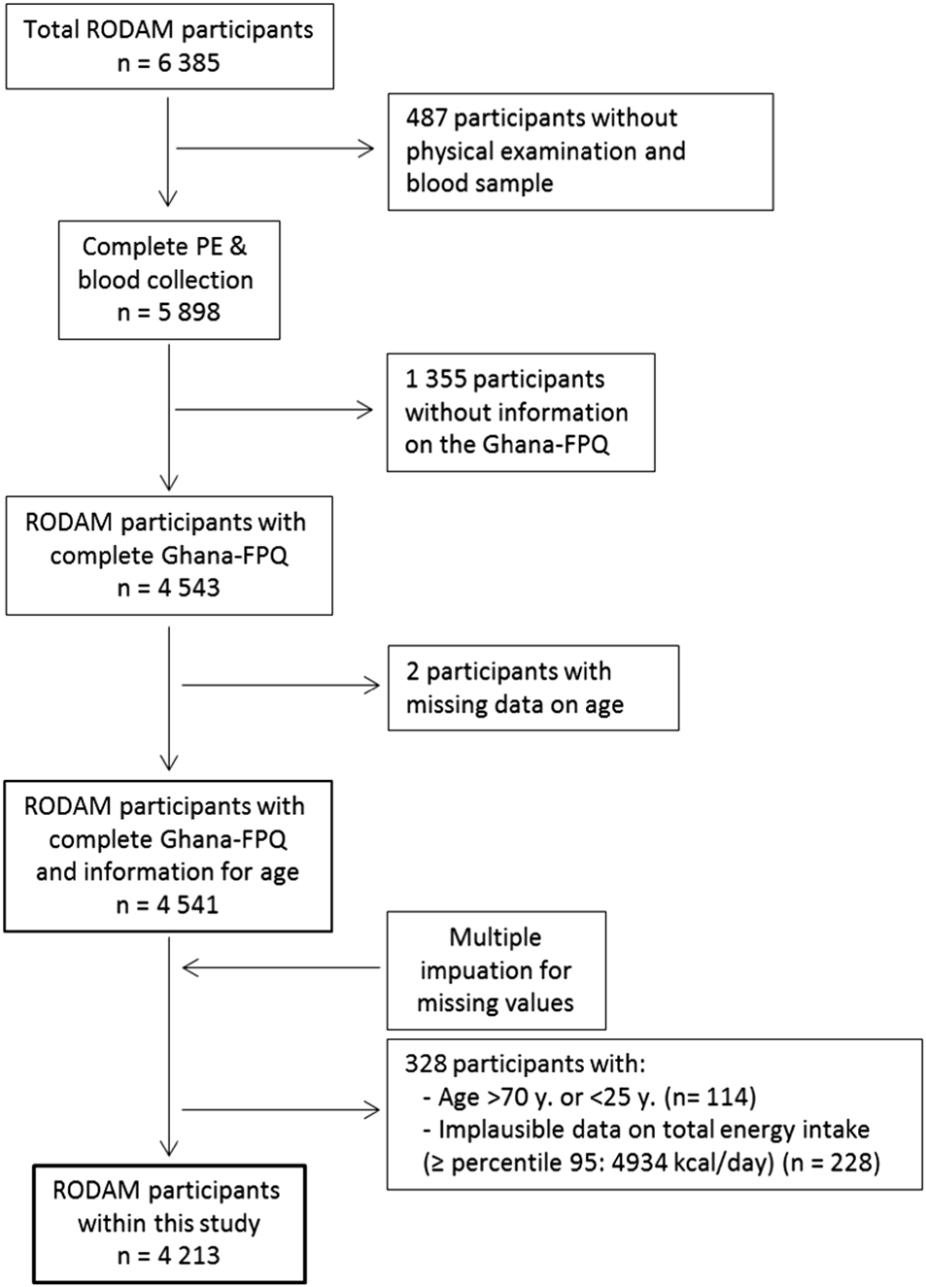
Flow-chart of excluded RODAM study participants, because of missing or implausible data. The exclusion of those participants with total energy intake >percentile 95 (4934 kcal per day) allowed to control for normality. The 1355 participants without information on the Ghana-FPQ comprise participants in who this was not conducted (*n* = 1262), and participants with the whole questionnaire or one or more whole sections blank (*n* = 93). FPQ Food Propensity Questionnaire, MI multiple imputation, PE physical examination

The detailed objectives and procedures of the multi-centre, cross-sectional RODAM study have been published elsewhere^13^. In brief, 6385 Ghanaian adults aged ≥18 years from urban (Kumasi and Obuasi, located in the Ashanti Region) and rural areas of the same region in Ghana, and residents in Amsterdam, London and Berlin, were recruited. The majority of Ghanaian migrants in Europe originated from the Ashanti Region and were first-generation migrants. The primary aim of the RODAM study was to identify the relative contributions of non-genetic and (epi)genetic risk factors for obesity and T2D in this population of West African origin. Data collection comprised a general questionnaire and a detailed dietary assessment. Furthermore, physical examinations were conducted and biological samples (fasting blood) were collected. Participants were instructed to remain fasting from 10:00 p.m. on the evening before the examination day and to abstain from alcoholic drinks, smoking and excessive physical activity. Recruitment strategies were adapted to the local circumstances at the various sites; in Ghana, census data of 2010 were used to draw rural and urban participants in the Ashanti Region. In Amsterdam, the Municipal Health Register was used to randomly select Ghanaian migrants who were invited by postal mail and home visits. In London, Ghanaian organizations were contacted and lists of these organizations were obtained from the Ghanaian Embassy and the Association of Ghanaian churches in the United Kingdom. In Berlin, the registration office of the federal state of Berlin provided a list of Ghanaian individuals, but due to the low response to written invitations, member lists of Ghanaian churches and organizations served as the sampling frame. The response rates were 76% in rural Ghana and 74% in urban Ghana. In Amsterdam, 67% replied, and of these, 53% agreed and participated. In London, of those individuals who were invited based on their registration in Ghanaian organizations, 75% agreed and participated in the study. In Berlin, this figure was 68%. Ethical approval was obtained from the local ethics committees at all study sites and all participants gave informed written consent. Figure 1 presents the flow diagram for the current analyses after multiple imputation of missing values and further exclusion of participants because of implausible data. This resulted in a sample size of 4543 participants for the characterization of dietary behaviour and a sample size of 4213 participants for the examination of the diet–disease associations.

### Nutritional assessment and DPs

The methods of dietary assessment have previously been described in detail^12^. In summary, at all RODAM study sites, food intake was assessed with a standardized semi-quantitative Food Propensity Questionnaire (Ghana-FPQ). The Ghana-FPQ queries for the usual intake frequencies of 134 food items in the preceding 12 months. Due to the semi-quantitative nature of the Ghana-FPQ, there was a lack of information on portion sizes for some of the included food items. Thus, 24h dietary recalls were conducted in a random sub-sample in each study site (*n* = 251) in order to estimate portion sizes as well as nutrient content of certain recipes. Lastly, the German Nutrient Database (BLS 3.01) (2010) and the West African Food Composition Table (2012) were used to translate usual food intake into total energy (kcal per day), and macro-nutrient and micro-nutrient intakes^12^.

A detailed description of the DPs extraction has also previously been published^12^. Briefly, the 134 food items included in the Ghana-FPQ were grouped into 30 food groups according to their nutrient profile and culinary use. Then, DPs were identified by means of principal component analysis (PCA) using the PROC FACTOR procedure in SAS 9.4. This method identified principal components that explain the maximum of the total variance of food intake. The factors were orthogonally rotated (varimax rotation) to ensure that these remained uncorrelated, facilitating their interpretability. Three DPs were identified; the “mixed” DP explained 14.4% of the total variance in food intake; the “rice, pasta, meat and fish” DP explained 8.8%; and “roots, tubers and plantain” DP explained 5.7% of the total variance in food intake. Every participant was assigned a score for each of the identified DPs to rank the participants according to DP adherence; higher scores were translated into higher adherence. As previously described^12^, these three main DPs were highly site-specific. Therefore, in the present work, we additionally derived two DPs per each study site for a more comprehensive and accurate examination of the diet–T2D associations, using the identical procedures.

### Assessment of T2D

Fasting blood samples were collected according to standard procedures by trained research assistants at the time of the physical examination. Samples were processed and aliquoted right after collection and stored temporarily at −20 °C and then transported to the local research centres where these were stored at −80 °C. Later, the samples were shipped to the analytical laboratory (Charité, Berlin) for the measurement of the biochemical parameters. Fasting plasma glucose was measured in venous blood (fluoride; ABX Pentra 400 chemistry analyser; HORIBA ABX SAS, Montpellier, France). T2D was defined according to the World Health Organization (WHO) diagnostic criteria (fasting plasma glucose ≥7.0 mmol/L, or current use of medication prescribed to treat diabetes, or self-reported diabetes)^14^.

### Assessment of covariates

Every participant completed a general questionnaire about socio-demographics and lifestyle. This was applied in face-to-face interviews by trained study personnel or self-administered based on the preference of the participants. Educational status assessment was adapted to local circumstances and comprised four categories: never been to school or elementary school; lower vocational schooling or lower secondary schooling; intermediate vocational schooling or intermediate/higher secondary schooling and; higher vocational schooling or university. The WHO STEPwise approach to chronic disease risk Surveillance (STEPS) Questionnaire^15^ was used to measure physical activity (METs-h per week), which included physical activity at work, while commuting and in leisure time. Smoking status was assessed through the question “Do you smoke at all?” and participants were categorized as current, former or non-smokers. Height (cm) was measured with a portable stadiometer, weight (kg) with a digital scale and waist circumference (cm) with a measuring tape (all devices SECA, Germany). Body mass index (BMI) was calculated as weight/height^2^ (kg/m^2^).

### Statistical analysis

As shown in Fig. 1, from the 4543 RODAM study participants with the physical examination, with Ghana-FPQ data and with blood samples, two participants were excluded for missing data on age. From the remaining 4541 participants, regression-based multiple imputations were conducted to impute missing information on the covariates considered in the analyses (education, total energy intake, smoking, physical activity, BMI, waist circumference). Five imputed datasets were created. For the main analysis on the associations of the DPs with T2D, missing values in the covariates were observed in 10.6% of participants. After multiple imputations, participants aged <25 and >70 years were excluded as well as those with an estimated total energy intake >95th percentile (4394 kcal per day).

For the final RODAM study population (*n* = 4213), socio-demographic, anthropometric, lifestyle characteristics, and estimated total energy consumption were presented as mean (±standard deviation, SD) for normally distributed, continuous variables, and as median (IQR: interquartile range) for non-normally distributed, continuous variables. Categorical variables were presented as percentages.

For the associations of the DPs with T2D, multiple-adjusted logistic regression models (odds ratios (OR), 95% confidence intervals (CI), *p* values) were calculated. We constructed tertiles of adherence to the DPs and used the lowest tertile as the reference. Trend tests were calculated by modelling the median score of each tertile of adherence as a continuous variable. OR (95% CI) per 1 SD increase of the DP scores were also calculated. Model 1 was adjusted for age, sex and study site (rural Ghana, urban Ghana, Amsterdam, London, and Berlin, only in the case of the overall DP associations). Model 2 included further adjustment for educational status (never or elementary; low; intermediate; high vocational), total energy intake (kcal per day), smoking status (never; current/former) and physical activity (METs-h/week), and model 3 additionally included BMI (kg/m^2^) and waist circumference (cm). The residual method was used to obtain waist circumference adjusted for BMI. Interactions for the DPs–T2D associations with sex and with study site (considered as rural Ghana, urban Ghana and Europe) were calculated by evaluating the significance of the cross-product terms in the fully adjusted model.

### Sensitivity analyses

There is the possibility of reverse causation, namely that individuals with T2D might have changed their diet after the diagnosis. Therefore, sensitivity analyses were performed. The associations between the three main DPs and T2D were evaluated by restricting the analysis to screen-detected T2D cases. Due to sample size reasons, this sensitivity analysis was performed solely in the total study population.

### Code availability

SAS code for the primary analysis is available from the authors.

## RESULTS

### Study population

The general characteristics of the RODAM study population have already been described in detail [2]. The major socio-demographic, lifestyle and anthropometric characteristics are presented in Table 1. In the RODAM study population, the majority was female (62%) and middle-aged (mean, 46.2 years; SD, 11.0 years). Most of the participants had low, elementary or no formal education. Men, in comparison to women, had a higher educational level and were more likely to be current or former smokers (19.7 vs. 3.2%). Men also had higher total energy intake and physical activity levels. While women presented with higher BMI and waist circumference than men, the prevalence of T2D was lower among women (8.4 vs. 11.1%). In urban Ghana, there was a higher percentage of women, and more men attended in Europe. Participants in Europe had a higher educational degree than their counterparts in urban and rural Ghana. Ghanaians in Europe were most likely to be current or former smokers, followed by rural Ghana. Also, those in Europe had a higher total energy intake, but this was similar in the participants living in rural Ghana. Participants in Europe also had higher BMI and waist circumference, followed by urban Ghana, and also Europe presented the higher prevalence of T2D: Europe 11.7%, urban Ghana 9.5% and rural Ghana 4.9%.

**Table 1:** General characteristics of the RODAM study participants (*n* = 4213)

	All (*n* = 4213)	Men (*n* = 1572)	Women (*n* = 2641)	Rural Ghana (*n* = 942)	Urban Ghana (*n* = 1420)	Europe (*n* = 1851)
Sex (% male)	38.0	—	—	38.7	28.8	43.3
Age (years)	46.2 ± 11.0	47.0 ± 11.3	45.8 ± 10.8	46.8 ± 12.7	45.3 ± 11.4	46.7 ± 9.6
Education (%)						
Never or elementary	37.4	22.1	46.4	56.9	43.6	22.7
Low	37.5	41.4	35.1	31.9	39.0	39.1
Intermediate	16.5	22.5	12.9	7.6	12.7	23.9
Higher vocational	8.7	14.0	5.6	3.6	4.8	14.3
Smoking (% current or former)	9.4	19.7	3.2	7.9	6.9	12.0
Total energy intake (kcal per day)	2532 ± 838	2626 ± 862	2476 ± 818	2620 ± 842	2313 ± 668	2654 ± 916
Physical activity (METs-h per week)*	72 (14, 170)	96 (28, 198)	60 (10, 156)	90 (36, 168)	60 (6, 156)	64 (14,188)
BMI (kg/m^2^)	26.8 ± 5.5	24.9 ± 4.4	27.9 ± 5.7	22.6 ± 4.3	26.9 ± 5.3	28.8 ± 4.9
Waist circumference (cm)	89.7 ± 12.6	87.0 ± 12.2	91.3 ± 12.6	81.2 ± 10.8	89.3 ± 11.8	94.4 ± 11.6
T2D^‡^ (%)	9.4	11.1	8.4	4.9	9.5	11.7

Data are shown as mean ± standard deviation, unless otherwise stated

* Data are shown as median (percentile 25, percentile 75). ^‡^ T2D was defined according to the WHO diagnostic criteria (fasting plasma glucose ≥7.0 mmol/L, or current use of medication prescribed to treat diabetes, or self-reported diabetes). In this sub-sample of the RODAM study, 397 participants were diabetic. From them 338 participants presented fasting blood glucose values ≥7 mmol/L, 201 reported the use of tablets for diabetes and 259 self-reported to be diabetic

*BMI* body mass index, *T2D* type 2 diabetes, *MET* Metabolic Equivalent of Task, *RODAM* Research on Obesity and Diabetes among African Migrants, *WHO* World Health Organization

### Overall and site-specific DPs

The general characteristics of the three overall DPs were previously described in detail^12^. In summary, the “mixed” DP was correlated with high intakes of whole grain cereals, sweet spreads, dairy products, potatoes, vegetables, poultry, coffee and tea, sodas and juices, olive oil, other oils and margarines. This DP explained 14.4% of total variance of food intake. Moreover, RODAM participants adhering to this pattern were more likely to be women and current or former smokers, to have a higher education and to live in Europe. The second retained pattern was named “rice, pasta, meat and fish” and explained 8.8% of the total variance. This was positively correlated with the intakes of legumes, rice and pasta, egg, red meat, processed meat, fish, meaty mixed dishes, cakes and sweets, sodas and juices and condiments. Independent determinants of adherence to this pattern were male gender, younger age, current or former smoking, higher education, higher physical activity levels and urban Ghanaian residence. Lastly, the third DP identified was labelled as “roots, tubers and plantain” pattern and explained 5.7% of the total variance of food intake. This was correlated with higher intakes of refined cereals, fruits, nuts and seeds, roots, tubers and plantain, fermented maize products, legumes and palm oil. Participants with a higher adherence to this pattern were more likely to be based in rural Ghana.

**Fig. 2:**
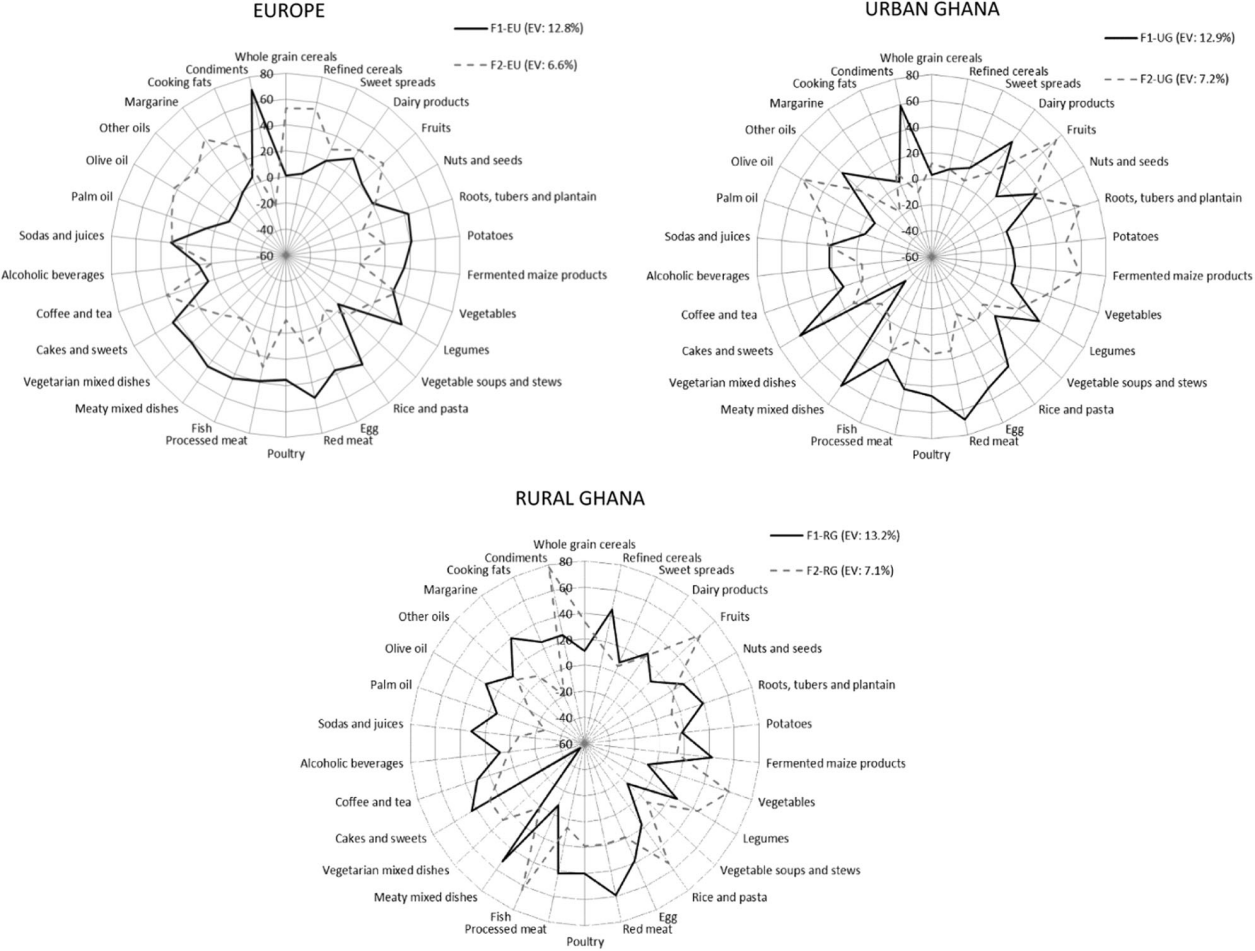
Site-specific dietary patterns derived by principal component analysis and rotated factor loadings. Food groups and their rotated factors loadings are shown. EV, explained variance (%)

Besides the three overall PCA-derived DPs, we extracted two DPs in each of the study sites (rural Ghana, urban Ghana and Europe) (Fig. 2). The Factor 1 observed in Europe (F1-EU) was characterized by high intakes of roots, tubers and plantain, potatoes, legumes, rice and pasta, red meat, processed meat, fish, meaty mixed dishes, vegetarian dishes, cakes and sweets and condiments. This factor explained 12.8% of the total variance in food intake in Europe. The Factor 2 retained in Europe (F2-EU) explained 6.6% of the variance and the characterizing food groups were whole grain cereals, refined cereals, dairy products, fruits, coffee and tea, olive oil and margarine. In urban Ghana, Factor 1 (F1-UG) explained 12.9% of the total variance in food intake. F1-UG was characterized by intakes of dairy products, legumes, rice and pasta, egg, red meat, poultry, processed meat, meaty mixed dishes, cakes and sweets and condiments. The second factor identified, Factor 2 (F2-UG), presented high factor loadings for fruits, roots, tubers and plantain, potatoes, fermented maize products and olive oil. F2-UG explained 7.2% of the total variance in food intake in urban Ghana. Lastly, Factor 1 in rural Ghana (F1-RG), explaining 13.2% of the total variance, was correlated with seldom intakes of vegetarian mixed dishes and frequent consumptions of refined cereals, roots, tubers and plantain, fermented maize products, egg, red meat, poultry, processed meat, meaty mixed dishes, cakes and sweets and margarine. Factor 2 in rural Ghana (F2-RG) was characterized by the intakes of fruits, vegetables, legumes, rice and pasta, fish and condiments. F2-RG explained 7.1% of the total variance in food intake in rural Ghana.

These newly extracted site-specific DPs presented some similarities with the overall previously identified DPs. The most prominent similarity was observed between the “rice, pasta, meat and fish” DP and the F1-UG in urban Ghana, which correlated positively with the intakes of legumes, rice and pasta, egg, red meat, processed meat, meaty mixed dishes and cakes and sweets.

### DPs and T2D

We calculated cross-sectional associations of the previously identified overall DPs in the total RODAM study population with T2D (Table 2). There was no association of the “mixed” DP with T2D, and this was also true for the “roots, tubers and plantain” DP. In contrast, higher adherence to the “rice, pasta, meat and fish” DP was associated with reduced odds of T2D. The fully multiple-adjusted model showed that participants in the highest tertile of this DP had a 34% lower chance of having T2D than those in the lowest tertile of DP adherence (OR_T3 vs. T1_: 0.66, 95% CI: 0.48–0.90). No significant interaction was observed between any of the three main DPs and sex or site (considered as rural Ghana, urban Ghana and Europe), thus stratified analyses were not calculated.

**Table 2:** ORs and 95% CIs for type 2 diabetes through tertiles of the dietary patterns’ scores and per 1 SD of the dietary pattern scores (*n* = 4213)

	Tertile 1	Tertile 2		Tertile 3		*p* for trend	per 1 SD	
	Ref.	OR	95% CI	OR	95% CI		OR	95% CI
“Mixed” dietary pattern								
Diabetes/no diabetes	115/1289	120/1285		162/1242				
Model 1	1.00	0.89	0.64–1.22	0.77	0.49–1.21	0.279	0.86	0.73–1.03
Model 2	1.00	0.93	0.67–1.29	0.92	0.57–1.48	0.815	0.95	0.78–1.15
Model 3	1.00	0.93	0.67–1.29	1.00	0.62–1.61	0.874	1.02	0.83–1.24
Screen-detecteddiabetes/no diabetes*	46/1272	52/1266		40/1278				
Model 3	1.00	1.14	0.72–1.82	0.87	0.41–1.85	0.617	0.80	0.56–1.13
“Rice, pasta, meat and fish” pattern								
Diabetes/no diabetes	176/1228	128/1277		93/1311				
Model 1	1.00	0.78	0.61–1.00	0.65	0.49–0.86	0.003	0.80	0.71–0.91
Model 2	1.00	0.81	0.63–1.05	0.74	0.55–1.01	< 0.001	0.85	0.74–0.97
Model 3	1.00	0.74	0.57–0.97	0.66	0.48–0.90	0.007	0.80	0.70–0.92
Screen-detected diabetes/no diabetes*	54/1264	48/1270		36/1282				
Model 3	1.00	0.88	0.58–1.34	0.70	0.42–1.16	0.171	0.85	0.68–1.06
“Roots, tubers, and plantain” pattern								
Diabetes/no diabetes	155/1249	139/1266		103/1301				
Model 1	1.00	0.96	0.74–1.24	0.82	0.61–1.10	0.180	0.93	0.81–1.06
Model 2	1.00	1.00	0.77–1.30	0.94	0.69–1.29	0.715	1.02	0.88–1.18
Model 3	1.00	1.00	0.77–1.31	0.98	0.71–1.35	0.900	1.04	0.90–1.21
Screen-detected diabetes/no diabetes*	59/1259	45/1273		34/1284				
Model 3	1.00	0.66	0.43–1.02	0.52	0.31–0.88	0.016	0.88	0.69–1.12

Model 1: age, sex, study site (5 study sites)

Model 2: Model 1 plus education (never or elementary; low; intermediate; high vocational), total energy intake (kcal per day), smoking (never; former; current), physical activity (METs-h per week)

Model 3: Model 2 plus BMI (kg/m^2^), waist circumference (cm)

*The sensitivity analyses were performed by excluding those with self-reported diabetes at the time of the physical examination (final *n* = 3954) and the adjustment set is the same as in Model 3

*OR* odds ratio, *CI* confidence interval, *SD* standard deviation

The sensitivity analyses revealed an inverse association of the “roots, tubers and plantain” DP with T2D when considering only screen-detected T2D cases as the outcome (OR_T3 vs. T1_: 0.52, 95% CI: 0.31–0.88; *p* for trend = 0.016) (Table 2). For the “rice, pasta, meat and fish” DP, the reduced sample size rendered the association with T2D non-significant. Still, the trend for an inverse association remained (OR_per 1 SD_: 0.85; 95% CI: 0.68–1.06). No association was observed for the “mixed” DP.

For site-specific DPs, the F1-UG pattern (Table 3) with similarities to the overall “rice, pasta, meat and fish” DP was associated with lower odds of T2D (OR_T3 vs T1_: 0.55, 95% CI: 0.30–0.98; *p* for trend = 0.027). Within Europe, F1-EUshared many features with the “rice, pasta, meat and fish” DP, and was also inversely associated with T2D (OR_T3 vs. T1_: 0.70, 95% CI: 0.47–1.03; *p* for trend = 0.058). Yet, none of the remaining site-specific DPs were related with T2D. After the exclusion of self-reported diabetes, the numbers of participants with T2D in each study site were too small to perform site-specific sensitivity analyses.

**Table 3:** ORs and 95% CIs for type 2 diabetes across tertiles of the dietary patterns’ scores and per 1 SD of the dietary patterns' scores

	Tertile 1	Tertile 2		Tertile 3		*p* for trend	per 1 SD	
	Ref.	OR	95% CI	OR	95% CI		OR	95% CI
Europe (*n* = 1851)								
Factor 1								
Diabetes/no diabetes	94/523	63/554		59/558				
Model 1	1.00	0.69	0.49–0.98	0.71	0.49–1.01	0.055	0.88	0.76–1.03
Model 2	1.00	0.72	0.50–1.02	0.79	0.54–1.16	0.204	0.94	0.79–1.11
Model 3	1.00	0.62	0.43–0.90	0.70	0.47–1.03	0.058	0.89	0.75–1.06
Factor 2								
Diabetes/no diabetes	69/548	82/535		65/552				
Model 1	1.00	1.07	0.75–1.53	0.71	0.49–1.04	0.051	0.95	0.82–1.10
Model 2	1.00	1.11	0.77–1.59	0.78	0.52–1.16	0.175	1.00	0.85–1.18
Model 3	1.00	1.11	0.77–1.61	0.94	0.62–1.42	0.716	1.09	0.92–1.29
Urban Ghana (*n* = 1420)								
Factor 1								
Diabetes/no diabetes	72/401	36/438		27/446				
Model 1	1.00	0.57	0.37–0.88	0.53	0.32–0.86	0.007	0.74	0.60–0.93
Model 2	1.00	0.58	0.37–0.91	0.58	0.33–1.05	0.045	0.77	0.59–1.02
Model 3	1.00	0.55	0.34–0.87	0.55	0.30–0.98	0.027	0.74	0.56–0.97
Factor 2								
Diabetes/no diabetes	42/431	50/424		43/430				
Model 1	1.00	1.11	0.71–1.72	0.99	0.63–1.57	0.941	0.98	0.82–1.18
Model 2	1.00	1.12	0.71–1.79	1.14	0.67–1.94	0.644	1.07	0.85–1.34
Model 3	1.00	1.13	0.71–1.81	1.17	0.68–2.01	0.575	1.08	0.87–1.36
Rural Ghana (*n* = 942)								
Factor 1								
Diabetes/no diabetes	20/294	19/295		7/307				
Model 1	1.00	1.18	0.61–2.31	0.47	0.19–1.15	0.103	0.81	0.57–1.15
Model 2	1.00	1.39	0.70–2.78	0.70	0.27–1.80	0.533	0.98	0.67–1.45
Model 3	1.00	1.60	0.79–3.26	0.69	0.26–1.83	0.557	0.99	0.67–1.48
Factor 2								
Diabetes/no diabetes	24/290	10/304		12/302				
Model 1	1.00	0.45	0.21–0.98	0.62	0.30–1.27	0.185	0.75	0.53–1.05
Model 2	1.00	0.45	0.20–0.98	0.81	0.35–1.89	0.518	0.81	0.54–1.20
Model 3	1.00	0.45	0.20–1.02	0.76	0.32–1.79	0.459	0.79	0.52–1.19

Model 1: age, sex

Model 2: Model 1 plus education (never or elementary; low; intermediate; high vocational), total energy intake (kcal/day), smoking (never; current; former), physical activity (METs-h per week);

Model 3: Model 2 plus BMI (kg/m^2^), waist circumference (cm)

*OR* odds ratio, *CI* confidence interval, *SD* standard deviation

## DISCUSSION

The present work describes cross-sectional associations of three previously identified DPs with T2D in the multi-centre RODAM study. We observed that a higher adherence to the “rice, pasta, meat and fish” DP was associated with lower odds of T2D. On the other hand, the “mixed” DP and the “roots, tubers and plantain” DP were not associated with T2D in the main analyses. However, after the exclusion of participants with self-reported diabetes, higher adherence to the “roots, tubers and plantain” DP was inversely associated with T2D. Moreover, the extraction of site-specific DPs confirmed the presence of DPs in Europe and in urban Ghana that resembled the “rice, pasta, meat and fish” DP. These two DPs were also associated with lower odds of T2D.

The RODAM study, with its particular design, presents the perfect frame to study dietary behaviour and the nutrition transition in West African populations living in their country of origin and in Europe. In our previous work, we identified three overall DPs among Ghanaian adults that agreed with the nutrition transition theory^12^. Indeed, the indigenous West African foods continued to be consumed at all study sites, while there was a trend towards increased intakes of westernized foods paralleling urbanization. At the same time, adherences to the “mixed” DP and the “rice, pasta, meat and fish” DP were strongly driven by higher education. With regard to locally specific dietary habits, westernized food items characterized the F1-EU and the F1-UG. Beside these similarities with the “rice, pasta, meat and fish” DP, the F1-EU was also correlated with the intakes of traditional foods, including roots, tubers, plantain and fermented maize products. At the same time, the F2-EU positively correlated with the intakes of grains, dairy products, fruits, coffee and tea, olive oil and margarine, resembling a “breakfast” DP.

Similarly, for urban Ghana, F1-UG reflected westernized dietary behaviour, while F2-UG strongly correlated with typical Ghanaian foods. These results agree with our previous observations in an independent urban Ghanaian population^16^. A “purchase” DP that was also characterized by fresh market foods and ready-made groceries was identified, and a “traditional” DP that correlated with the indigenous Ghanaian foods was extracted^16^. For rural Ghana, both identified patterns argue against an ongoing nutrition transition in rural areas. Rather, the F1-RG represents a meat-based traditional DP, while the F2-RG reflects a fish- and vegetable-based indigenous DP.

### DPs and T2D

The investigation of the associations between DPs and T2D in African population is scarce. In the present study, the “rice, pasta meat and fish” pattern was associated with lower odds of T2D, and so did the F1-EU and the F1-UG. The composition of these patterns, per se, does not suggest a beneficial effect on health; these correlated with the intakes of meat and processed meat as well as cakes and sweet foods previously described to have a negative health effect, particularly for T2D^17–19^. However, other items with potentially beneficial effects are also present in this pattern, such as legumes and fish^20–22^. A plausible explanation to this observation could be the consumed amounts of these food items, for which the DP analysis does not provide information. Indeed, the worldwide statistics reported that the consumption of fish and legumes in Ghana exceeds the one in Germany by far, whereas this is the opposite for meat and meat products^23^. On the other hand, a work conducted within the RODAM population observed that the “rice, pasta, meat and fish” DP was positively correlated with health-beneficial food variety, which could partially explain the associations observed^24^. Moreover, our observations are also in line with the work of Frank et al.^16^ above mentioned. In this work, also the “purchase” DP was associated with lower odds of T2D^16^. And in further analysis it was observed that this “purchase” DP was characterized by a health-beneficial serum phospholipid fatty acid profile^25^. These results suggest that further analyses are required to understand the observed findings and that food intake in this specific population differs much from our knowledge on host European population.

Most interestingly, the “roots, tubers and plantain” DP exhibited an inverse association with T2D after the exclusion of subjects with self-reported diabetes. Beside the possibility of reverse causation, this implies a health-beneficial effect of the traditional-oriented dietary behaviour. This pattern was characterized by refined cereals, fruits, nuts and seeds, roots, tubers and plantain, fermented maize products, legumes, palm oil and condiments. In fact, many of these food items have been attributed to health-beneficial effects, like fruits, nuts and seeds^26, 27^. In the case of the typical fermented products (banku and kenkey), a study from Malawi showed that fermented maize products have a lower glycaemic index than other maize flour^28^.

### Strengths and limitations

Some strengths and limitations concerning the present work must be mentioned for careful interpretation of our results. The first and main limitation is the cross-sectional design of the RODAM study that limits the possibility of establishing diet–T2D causal relationships. Also, the Ghana-FPQ has not been validated yet and this can have measurement error, which could lead to substantial bias in later analyses^29^. Nevertheless, this tool has been culturally adapted for Ghanaian populations and we have used highly standardized methods and measurement procedures in all sites, which allowed the collection of homogeneous information. Of note, screen-detected T2D for the outcome definition in this study does not substitute the medical diagnosis of T2D to initiate treatment. Furthermore, we have adjusted our analysis for the available information on several dietary and non-dietary variables, allowing us to control for possible confounding. Most importantly, we included measures of general and central adiposity in the regression models to account for potential effect mediation. Yet, the role of the sexual dimorphism for the relationship between general obesity and T2D remains to be clarified. On the other hand, the multi-center design of the RODAM study is its most remarkable strength. This genetically homogenous population of Ghanaians living in different settings in Africa and Europe used the same measurement procedures in all sites collecting high-quality and comparable information^2^.

In the present work, we have observed that DPs characterized by the intakes in legumes and fish, but also in meat and confectionery may exert health-beneficial effects among Ghanaian adults, irrespective of their place of residence. On the other hand, the importance of a more traditional Ghanaian diet, rich in typical staples, vegetables and legumes, still remains unclear.

### Ethics statement

The RODAM Study was conducted according to the guidelines laid down in the 1964 Declaration of Helsinki and its later amendments. All procedures involving human subjects were reviewed and approved by the respective ethics committees in Ghana, the Netherlands, the UK and Germany. Written informed consent was obtained from all participants.
